# Lack of Differences in Inflammation and T Cell-Mediated Function between Young and Older Women with Obesity

**DOI:** 10.3390/nu12010237

**Published:** 2020-01-16

**Authors:** Maria Carlota Dao, Edward Saltzman, Melissa Page, Jillian Reece, Tara Mojtahed, Dayong Wu, Simin Nikbin Meydani

**Affiliations:** 1 Jean Mayer USDA Human Nutrition Research Center on Aging, Tufts University, Boston, MA 02111, USA; edward.saltzman@tufts.edu (E.S.); tara418@gmail.com (T.M.); dayong.wu@tufts.edu (D.W.); simin.meydani@tufts.edu (S.N.M.); 2 Weight and Wellness Center, Tufts Medical Center, Boston, MA 02111, USA; page.melissamarie@gmail.com (M.P.); JReece1@tuftsmedicalcenter.org (J.R.)

**Keywords:** obesity, aging, T cell-mediated function, inflammation

## Abstract

Both obesity and aging are associated with dysregulated immune and inflammatory responses. There is limited knowledge, however, on differences in the immune system between young and older adults with obesity. The goal of this study was to compare circulating inflammatory cytokines and T cell-mediated immune response between young and older women with obesity. Twenty-three young (23–43 years) and 21 older (60–83 years) women with obesity were recruited at the Weight and Wellness Center at Tufts Medical Center. Circulating inflammatory cytokines (CRP, IL-6, and IL-1β) and ex vivo indicators of T cell-mediated immune function were compared between the groups. Older women with obesity had significantly fewer circulating CD3+, CD8+, CD19+, and natural killer T (NKT) cells compared to young women with obesity (*p* = 0.016, *p* < 0.0001, *p* = 0.0003, and *p* < 0.0001, respectively). However, with few exceptions, there was no significant difference in inflammation markers or stimulated lymphocyte proliferation and cytokine production by peripheral blood mononuclear cells between young and older participants. These findings are in contrast to those previously reported in young and old subjects with healthy weight and call for further investigation into the impact of obesity on premature aging of the immune system.

## 1. Introduction

The obesity epidemic is affecting older populations at an increasing rate. According to a report from NHANES (2007–2008), 69% of people older than 60 years have overweight or obesity (body mass index, BMI ≥ 25 kg/m^2^) [1]. In addition to a higher BMI, sarcopenia, or loss of lean body mass together with a gain in fat mass, frequently occurs with aging. Certain impairments seen in obese populations are comparable to those observed in the elderly, such as leptin resistance, chronic inflammation, and immune response deficiencies [2,3].

Several studies have shown that, compared to individuals with healthy weight, adults with obesity have a blunted lymphocyte response to mitogenic or antigenic stimulation, lower naïve lymphocyte output, a skewed T cell phenotype towards T helper 1 (Th1) cells, and impaired macrophage and natural killer (NK) cell function [4,5,6,7]. Numbers of certain types of lymphocytes, namely CD3+ cells, CD4+ and CD8+ T cell subsets, and B cells are depleted in individuals with obesity with respect to those with a healthy weight [8,9]. In both mice and humans, there is evidence that obesity induces premature thymic involution and decreased thymic output, a phenomenon similar to that seen in aging [10]. These deficiencies are thought to contribute to a higher risk of infections and chronic disease [7,11]. In fact, several of the impairments that occur with obesity resemble the immunodeficiency observed with aging, termed immunosenescence. With older age, the adaptive immune response becomes impaired, as seen by decreased antigen-induced lymphocyte proliferation, diminished number of naïve lymphocytes, a decline in function of CD4+ Th cells and CD8+ cytotoxic effector T cells, and dysregulation of cytokine production by CD4+ and CD8+ T cell subpopulations, all concomitant with thymic involution [12,13,14]. Innate immunity, including macrophage and neutrophil function, and NK cell cytotoxicity, is also impaired with aging [15]. This weakening of immune cell function with aging translates into higher risk of infections, reduced effectiveness in responding to newly encountered pathogens, and decreased response to vaccines, together with higher risk for chronic inflammatory conditions including autoimmune disease [15,16]. Furthermore, a low-grade, chronic inflammation develops with aging and obesity, as indicated by higher circulating concentrations of C-reactive protein (CRP) and interleukin 6 (IL-6) [6,14]. 

Despite these facts, there is limited evidence available on the impact of obesity on immune response in elderly populations. While there is evidence of premature immunosenescence in obese adult mice [17], a direct comparison between young and older adults with obesity has not been conducted, thus it is not known whether obesity induces further dysregulation of immune and inflammatory responses in older adults. 

In this pilot study, we compared in vivo markers of systemic inflammation and ex vivo parameters of T cell-mediated immunity between young (18–45 years) and older (>60 years) women with obesity. We selected immune response parameters and inflammatory cytokines previously reported to be impacted by aging, including peripheral blood mononuclear cell (PBMC) subpopulations, lymphocyte proliferation, cytokine production by ex vivo stimulated PBMCs, and pro-inflammatory cytokine profiles in serum. 

## 2. Materials and Methods

### 2.1. Study Population and Sample Collection

This was a cross-sectional analysis in young (18–45 years) and older (>60 years) (BMI ≥ 30 kg/m^2^) women with obesity conducted at the Jean Mayer USDA Human Nutrition Research Center on Aging (HNRCA) at Tufts University in collaboration with the Weight and Wellness Center (WWC) at Tufts Medical Center. There were 23 participants in the YG and 21 in the OG. Study participants were recruited at the WWC, a medical and surgical weight loss clinic, prior to weight loss treatment. Exclusion criteria included pregnancy, or giving birth within six months prior to recruitment, reported weight loss of more than 3% within three months before recruitment, history of eating disorders, renal or hepatic disease, gastrointestinal disorders, prior gastric restrictive surgery, hematological malignancies, cancer, chronic infections, or autoimmune disease. This study was conducted according to the guidelines laid down in the Declaration of Helsinki and all procedures involving human subjects were approved by the Tufts Medical Center/Tufts University Institutional Review Board. Written informed consent was obtained from all subjects. This study has been registered in ClinicalTrials.gov (NCT01636635).

### 2.2. Anthropometric Measures and Clinical Parameters

Measures included height, weight, BMI, and waist circumference (WC). For WC, the average of three consecutive measurements was taken at the level of the iliac crest region. After an overnight fast, 30-mL of venous blood were collected into EDTA and serum separation tubes. Participants reported having no infections or illness, and not taking antibiotics or receiving vaccinations in the two weeks prior to blood collection. Further, participants reported not taking non-steroidal anti-inflammatory drugs or anti-histamine medications for 72 h prior to blood sample collection. 

A full chemistry profile and complete blood count were measured for each participant. Serum CRP was measured with the high sensitivity IMMULITE-100 immunoassay (Siemens Healthcare Diagnostics, Los Angeles, CA, USA). Leptin, adiponectin, IL-6, and IL-1β were measured using multi-spot electrochemiluminescence assays (Meso Scale Discovery, Gaithersbug, MD, USA).

### 2.3. PBMC Isolation

All cell preparation and culture work were conducted under sterile conditions. Blood was collected into EDTA tubes and PBMC were isolated with gradient centrifugation using a Histopaque matrix (Sigma-Aldrich, St. Louis, MO, USA). RPMI 1640 media supplemented with HEPES (25 mmol/L), glutamine (2 mmol/L), and 100 kU/L penicillin/100 mg/L streptomycin (Gibco) was used for PBMC isolation, and 1× PBS was used in washing steps. After the last wash, PBMC were suspended at a density of 20 million cells/mL. Cells from this suspension were used for all assays. Flow cytometry analysis was conducted in fresh PBMC (25,000 cells per sample). All cell cultures were incubated at 37 °C, 5% CO_2_, and 95% humidity in media supplemented with 5% heat-inactivated fetal bovine serum (FBS) or autologous human serum (HS), as specified.

### 2.4. PBMC Subpopulations 

Percentages of PBMC subpopulations were determined using flow cytometry with BD Biosciences reagents and protocols (BD Biosciences, San Jose, CA, USA). Antibodies against the following surface markers were used: CD3, CD4, CD8, CD19, NKG2D, and CD14 (eBioscience, San Diego, CA, USA). NKT (natural killer T) cells were identified as co-expressing NKG2D and CD3. Fixed cells were analyzed with an Accuri C6 flow cytometer (BD Biosciences, San Jose, CA, USA) and FlowJo Software version 10 (Tree Star, Inc., Ashland, OR, USA). Isotype controls for each antibody class and fluorochrome were used as negative controls. The numbers of PBMC subpopulations (number of cells/µL blood) were calculated as follows: Total number of PBMC was determined from the %PBMC (%lymphocytes + %monocytes) of white blood cells from CBC analysis. Then, the number of each subpopulation was determined from the %subpopulation of PBMC (as measured by flow cytometry).

### 2.5. Lymphocyte Proliferation

Lymphocyte proliferation was assessed by [3H]-thymidine incorporation in cells that were treated with antibodies against CD3 (T cell receptor) and CD28 (T cell co-receptor) (anti-CD3/CD28, eBioscience) or T cell mitogen phytohemagglutinin (PHA) (Difco Laboratories), and cultured in 5% FBS or 5% HS. For anti-CD3/CD28 stimulation, 96-well round bottom cell culture plates were coated with serially diluted anti-CD3 in 1× PBS (1 µg/mL, 5 µg/mL, or 10 µg/mL), incubated for 2 h, and washed twice with sterile 1X PBS. Cells in 5% FBS or 5% HS at a density of 1 × 10^5^ cells/well, and anti-CD28 (2 µg/mL) were added to the coated wells. PHA (2 µg/mL, 5 µg/mL, or 25 µg/mL) was used for mitogenic stimulation. All samples were cultured in triplicate, and unstimulated controls were included for all subjects and cell culture conditions. After stimulation for 68 h, cells were pulsed with 0.5 µCi [3H]-thymidine/well (Perkin Elmer, Waltham, MA, USA) and cultured for additional 4 h. Plates were then frozen at −80 °C for later analysis. Thawed plates were trypsinized for 1 h and harvested onto glass fiber mats using a cell harvester (Perkin Elmer, Waltham, MA, USA). Incorporation of [3H]-thymidine was determined by liquid scintillation counting using a Micro Beta 2 MicroPlate counter (Perkin Elmer, Waltham, MA, USA). Results, expressed as counts per minute (CPM), were used as a quantitative measurement of lymphocyte proliferation. 

### 2.6. Cytokine Production by Stimulated PBMC

PBMC were cultured in 5% FBS or HS at a density of 1 × 10^6^ cells/well in 24-well plates, and stimulated with anti-CD3/CD28 (5 µg/mL/2 µg/mL), PHA (10 µg/mL), or lipopolysaccharide (LPS) (10 ng/mL). Unstimulated controls were included for all culture conditions. After 72 h (anti-CD3/CD28 and PHA) or 24 h (LPS) of incubation, supernatant was harvested by centrifugation at 4 °C and stored at −80 °C for later analysis. IL-6, IL-1β, TNF-α, and IFN-γ were measured using multi-spot electrochemiluminescence assays (Meso Scale Discovery, Rockville, MD, USA). IL-2 was measured using an ELISA Kit (eBioscience, San Diego, CA, USA). Cytokines were undetectable in unstimulated samples and therefore not presented in the results.

### 2.7. Statistical Analysis

Number of participants per group was determined based on the study by Ahmed et al. [18], where weight loss through calorie restriction in adults resulted in a 63% increase in cell proliferation after stimulation with PHA. Thus, 22 subjects per group would lead to >80% power to detect a significant difference in cell proliferation at the 0.05 level. Variables with a skewed distribution are reported as median (Q1–Q3), and those with a normal distribution as mean ± SD. Wilcoxon rank sum test was used for all comparisons between the YG and the OG. Spearman, or partial Spearman, correlation analysis was used to determine the association between immune parameters and age or BMI. Sample size for each analysis is indicated in tables and figures. Statistical significance was set at alpha = 0.05. SAS 9.4 for Windows (SAS Institute, Cary, NC, USA) was used for all statistical analyses.

## 3. Results

### 3.1. Differences in Anthropometry and Inflammatory Cytokines between Young and Older Women with Obesity

The average age was 36 ± 6 years for the young group (YG, 23–43 years, N = 23) and 68 ± 8 years for the older group (OG, 60–83 years, N = 21) (Table 1). Both groups had obesity, and, although BMI (*p* = 0.004) and WC (*p* = 0.04) were significantly higher in the YG, there was substantial overlap in these measures between the groups. There were significantly higher numbers of white blood cells (*p* = 0.008) and platelets (*p* = 0.002) in the YG, while adiponectin was higher in the OG (*p* < 0.001, Table 1). Analysis of inflammatory cytokines in serum showed that IL-1β was significantly higher in the OG (*p* = 0.02). However, contrary to previous reports in healthy non-obese older adults compared to young adults [19,20], there was no significant difference in serum CRP or IL-6 concentration between YG and OG.

### 3.2. Comparisons in PBMC Subpopulations between Young and Older Women with Obesity within PBMC Subpopulations

There was a significantly higher percentage and density (expressed in number of cells/µL blood) of CD8+ (*p* = 0.002 for percentage and *p* < 0.0001 for number) and natural killer T (NKT) cells (*p* < 0.0001 for percentage and *p* < 0.0001 for number) in YG (Table 2), two cell types involved in the adaptive and innate cytotoxic responses, respectively. In YG, there was also a significantly larger percentage (*p* = 0.007) and number (*p* = 0.0003) of B cells, quantified as CD19+ cells, and a larger number of total T cells (*p* = 0.016), quantified as CD3+ cells. When considering percentages of PBMC subpopulations and adjusting for BMI, Spearman partial correlation analysis showed a significant association between age and CD8+ cells (r = −0.48, *p* = 0.001), NKT cells (r = −0.61, *p* < 0.0001), and CD19+ cells (r = −0.34, *p* = 0.03). Similarly, when considering density of PBMC subpopulations and adjusting for BMI, there was a significant partial Spearman correlation between age and CD3+ cells (r = −0.31, *p* = 0.05), CD8+ cells (r = −0.56, *p* = 0.0001), NKT cells (r = −0.68, *p* < 0.0001), and CD19+ cells (r = −0.45, *p* = 0.003). The OG had significantly more %CD4+ T cells than the YG (*p* = 0.02), but, when considering the number of CD4+ T cells per µL of blood, there was no difference between YG and OG. Other cell types in PBMC, namely NK cells (NKG2D+), or monocytes (CD14+, used as a marker of monocytes in humans), were not different between age groups.

### 3.3. Lymphocyte Proliferation in Young and Older Women with Obesity

**Figure 1:**
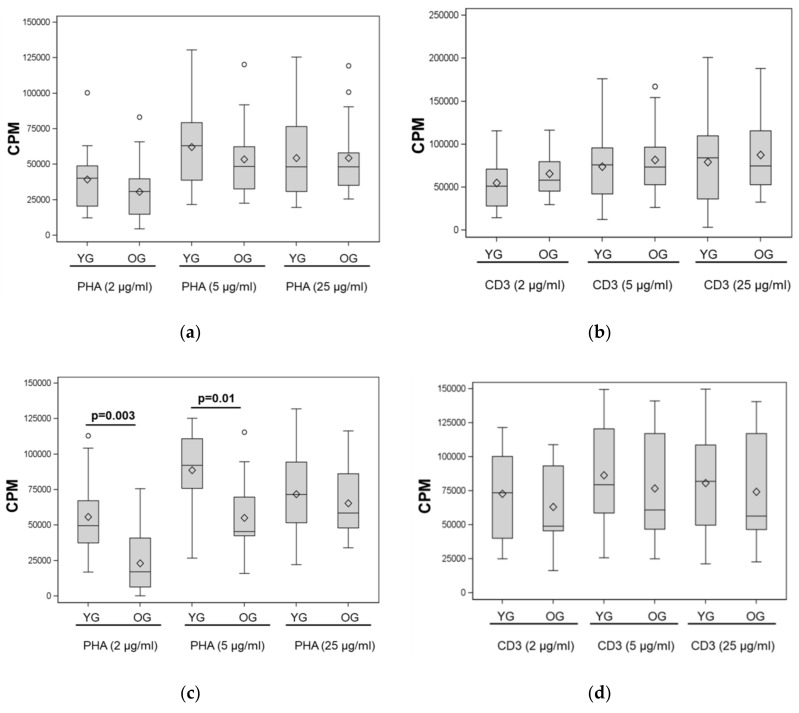
PHA- and anti-CD3/CD28-stimulated lymphocyte proliferation in young and older women with obesity. PBMC from young and older women with obesity were cultured in 5% FBS (**a**,**b**) or 5% autologous human serum (HS) (**c**,**d**) for 72 h in the presence of PHA (**a**,**c**) or anti-CD3/CD28 (**b**,**d**) at different concentrations. Medians and quartiles are shown in boxplot, means are represented by diamonds. Significance of differences were determined by Wilcoxon rank sum test. The dots on the figures indicate data points that are outside of the interquartile range.

Lymphocyte proliferation was measured in culture containing 5% FBS or HS in response to PHA or CD3/CD28 stimulation. Contrary to what has been previously observed in young and older subjects with healthy weight [4,5], there was no age-related difference in proliferation of lymphocytes cultured in FBS under any stimulation condition tested (Figure 1a,b). However, higher lymphocyte proliferation was found in the YG when PBMC were cultured in medium containing HS and stimulated with PHA (at 2 μg/mL and 5 μg/mL) (Figure 1c).

Partial Spearman correlation analysis adjusting for BMI showed no association between PBMC proliferation and age for the FBS culture conditions (data not shown). For PBMC cultured in HS, there was a significant inverse association between age and PBMC proliferation stimulated by PHA at 2 µg/mL (r = −0.51, *p* = 0.005) and at 5 µg/mL (r = −0.49, *p* = 0.006). These findings show an attenuation in differences in lymphocyte proliferation between young and older subjects with obesity, compared to marked differences observed in previous reports between young and older populations with healthy weight [4,5,16].

### 3.4. Cytokine Production in Stimulated PBMC

There were no differences in cytokine production by PBMC stimulated with PHA or anti-CD3/CD28 for 72 h, or with LPS for 24 h. The cytokines measured were IL-2, IFN-γ, and TNF-α in the 72-h cultures and IL-1β, IL-6, and TNF-α in the 24-h cultures (Table 3). There were two exceptions in these observations: IL-6 production was higher in OG upon PBMC stimulation with LPS in 5% FBS (*p* = 0.02), and IFN-γ production was higher in YG upon stimulation with anti-CD3/CD28 in 5% HS (*p* = 0.03). Similarly to the lymphocyte proliferation results, the differences observed in cytokine secretion between young and older subjects with obesity are attenuated compared to what has been previously observed in healthy weight populations.

## 4. Discussion

To our knowledge, this is the first study comparing immune and inflammatory responses between young and older women with obesity. The results show substantial similarities in markers for inflammatory status and T cell-mediated function between young and older subjects, in contrast to previous studies in women with a healthy weight status that have reported significant differences between age groups [5]. These results suggest that obesity is an important determinant of inflammation and T cell-mediated immune impairment, and may be inducing an early immunosenescence.

Other reports have found significantly lower lymphocyte proliferation in response to PHA or anti-CD3/CD28 stimulation in healthy weight elderly compared to young subjects [4,5,16] while we found no differences, with the exception of PHA stimulation in autologous serum. This suggests that obesity may be impairing the immune system of young individuals in a manner similar to immunosenescence, perhaps because of extended periods of obesity-induced chronic inflammation and alterations in hormonal profiles. The notion of obesity-induced immunosenescence has been previously shown in mice [17]. It is noteworthy that the proliferative responses reported in young subjects with a healthy weight are much higher [4] than those shown in young subjects with obesity in our study. Further, it is interesting to note that, while there was no difference in lymphocyte proliferation between young and older adults with obesity when cultured in FBS, culturing lymphocytes in autologous serum resulted in re-appearance of an age difference in proliferative response (Figure 1). These observations suggest that, in addition to inducing defects in T cells, obesity may induce additional changes in serum of the old, exacerbating those related to aging.

Serum pro-inflammatory cytokines are higher in subjects with obesity compared to subjects with healthy weight, but T cell response and cytokine production after stimulation are usually impaired [21]. While serum IL-1β levels were higher in OG compared to YG, we found no difference in serum CRP and IL-6 between the two groups. This is contrary to previous reports of higher IL-6 and CRP levels in elderly compared to young healthy weight subjects [14]. Adiponectin was significantly higher in the OG, and this adipokine is known to have an anti-inflammatory effect [22,23] that could be contributing to the observed attenuated differences in chronic inflammation between the young and older obese groups. In summary, these results suggest that T cell-mediated response may be impaired by obesity in young individuals to a larger degree than in older individuals so that the age-related differences are abrogated. In other words, while obesity induces premature aging of T cell-mediated response in young individuals, it may not further impair the already weakened T cell-mediated response of the aged. On the other hand, age-related inflammation may be further exacerbated by obesity.

CD8+ T cells and NKT cells are cytotoxic cells in adaptive and innate immune system, respectively. In this study, older subjects with obesity had lower numbers of these cell populations than young subjects with obesity. Given that Nieman et al. found no difference in CD8+ or NKT cell cytotoxic activity or numbers between individuals with healthy and obese weight [9], the difference we found is likely due to the impact of aging alone rather than obesity. Indeed, previous studies have reported that function of cytotoxic T cells decline with aging, which may contribute to the impaired defense against infection in the elderly [12,13,14,15].

A limitation of our study is that it did not include a group of young and older subjects with a healthy weight, which otherwise would have allowed us to have a better differentiation between age and obesity-induced changes in immune and inflammatory responses. We therefore present our results in the context of several previous studies that have separately addressed the effects of age and obesity on immunity. The observation that proliferative response in young subjects with obesity in our study is much lower than those generally reported in other studies and closer to those reported for older adults [4] supports our suggestion that obesity “ages” T cells. It has been demonstrated that estrogen deprivation may modulate cytokine production and immune response [24]. Given that postmenopausal women were included in this study, and that obesity is also associated with altered sex hormone signaling [25], hormonal changes as well as other mechanisms influenced by obesity and aging may have modified the outcomes presented in this manuscript.

It is widely recognized that the elderly have higher inflammation and lower T cell-mediated response compared to younger adults. It is unlikely that the lack of age-related differences between young and older adults with obesity observed in this study is due to the sample size, as previous studies have shown differences in similar parameters using comparable (20 subjects per group in the study by Douziech et al.) or even smaller (six subjects per group in the study by Meydani et al.) sample sizes and similar methodology [4,5].

In the current study, the YG had a significantly higher BMI and WC than the OG. However, there was no difference in circulating leptin. Given the increase in fat mass in aging, it is likely that the two groups had comparable fat mass. Future studies should include a comparison of body composition between groups and its relation to observed changes in immune response. Future studies should also include in vivo measures of the immune response to better understand the clinical significance of interaction between obesity and aging.

## 5. Conclusions

In summary, this study shows for the first time that patterns in inflammation and T-cell mediated immunity are similar between young and older women with obesity, and that young women with obesity have immune function parameters that bear many similarities to those previously linked to aging. These results raise important questions on the relative effects of obesity and chronological age on immune response. Future interventions should address whether immune function in persons with obesity is modifiable, e.g., through sustained weight loss [18], and the underlying mechanisms involved in the observed impairments linked to the obese state.

## Figures and Tables

**Table 1:** Subject Characteristics.

Variable	YG (N = 23)	OG (N = 21)	*p* *
Age (yeaers)	36 ± 6	68 ± 8	<0.0001
Race (N)			0.63
White	16	16	
Black	5	5	
Hispanic	2	0	
BMI (kg/m^2^)	43.3 ± 4.2	38.9 ± 5.0	0.004
Weight (kg)	118.3 ± 12.0	102.0 ± 13.7	0.0007
WC (cm)	121.2 ± 10.5	114.3 ± 10.5	0.04
Albumin (g/dL)	4.3 ± 0.3	4.3 ± 0.2	0.63
Total cholesterol (mg/dL)	188 ± 34	198 ± 32	0.28
Triglycerides (mg/dL)	96 (71−170)	100 (73−134)	0.88
Glucose (mg/dL)	96 (92−101)	96 (89−102)	0.98
White blood cells (×1000/µL)	7.8 ± 1.8	6.3 ± 1.5	0.008
Monocytes (%)	6.3 ± 1.0	6.7 ± 1.5	0.51
Neutrophils (%)	62 (55-65)	62 (59-65)	0.36
Lymphocytes (%)	30.3 ± 6.8	27.6 ± 5.6	0.40
Red blood cell (mil/µL)	4.7 ± 0.3	4.5 ± 0.5	0.07
Platelet count (×1000/µL)	313 ± 81	238 ± 60	0.002
Hemoglobin (g/dL)	13.5 (12.8−13.9)	13.1 (12.4−14.1)	0.74
Serum CRP (mg/L)	7.4 (4.3, 14.2)	3.6 (1.8, 12.6)	0.14
Serum IL-6 (pg/mL)	7.7 ± 3.8	9.3 ± 3.7	0.09
Serum IL-1β (pg/mL)	0.66 ± 0.40	0.94 ± 0.41	0.02
Serum Leptin (ng/mL)	454,476 ± 188,536	445,228 ± 200,414	0.94
Serum Adiponectin (μg/mL)	9568 ± 3014	16,034 ± 6675	0.0002

Young (YG) and older (OG) group. Mean ± SD or Median (Q1–Q3). * Significant differences determined by Wilcoxon rank sum test, and Fisher’s exact test for race. WC, waist circumference; CRP, C reactive protein; IL-6, interleukin 6; IL-1β, interleukin 1β; N, number; ×, times.

**Table 2:** PBMC composition in young and older women with obesity.

	Percentage of Total PBMC			Density (×1000/µL Blood)		
PBMC Subpopulation	YG (N = 22)	OG (N = 20)	*p* *	YG (N = 22)	OG (N = 20)	*p* *
CD3+	74 (62−76)	68 (63−75)	0.52	1.77 (1.48−2.24)	1.47 (1.07−1.74)	0.016
CD4+	55 (52−62)	63 (58−66)	0.02	1.52 (1.23−1.77)	1.3 (1.09−1.55)	0.10
CD8+	17 (16−22)	13 (10−16)	0.002	0.5 (0.36−0.60)	0.27 (0.19−0.35)	<0.0001
CD19+	9 (7−11)	6 (5−9)	0.007	0.29 (0.17−0.35)	0.13 (0.10−0.17)	0.0003
NK	8 (6−10)	8 (6−11)	0.91	0.21 (0.17−0.28)	0.15 (0.12−0.23)	0.08
NKT	16 (12−21)	8 (6−11)	<0.0001	0.43 (0.33−0.56)	0.16 (0.12−0.25)	<0.0001
CD14+	14 (9−17)	15 (11−18)	0.19	0.32 (0.26−0.54)	0.33 (0.24−0.46)	0.68

Young (YG) and older (OG) group. Median (Q1–Q3). * Significant differences determined by Wilcoxon rank sum test. PBMC, peripheral blood mononuclear cell; CD3, CD3+ subpopulation (includes T cells and NKT cells); CD4, CD4+ T cell; CD8, CD8+ T cell; CD19, marker for B cells; NK, natural killer cell; NKT, natural killer T cell (CD3+, NK+); marker for CD14, monocytes; N, number; ×, times.

**Table 3:** Ex vivo cytokine production by stimulated PBMC from young and older women with obesity.

Culture Condition	Stimuli	Cytokine (pg/mL)	YG (N = 22)	OG (N = 19)	*p* *
	**anti-CD3/CD28**	**IFN-γ**	47,742 (31,056−114,687)	30,115 (15,261−55,701)	0.20
		**IL-2**	14,562 (9972−19,535)	16,639 (7488−20,464)	1.00
		**TNF-α**	12,703 (7801−17,916)	13,645 (6989−16,113)	0.74
**FBS**	**PHA**	**IFN-γ**	8888 (6388−11,276)	10,886 (4685−14,881)	0.76
		**IL-2**	821 (145−1531)	1433 (431−3714)	0.30
		**TNF-α**	2240 (1427−4173)	3033 (1094−3671)	0.52
	**LPS**	**IL-1**	4308 (2974−5298)	3809 (2691−5381)	0.91
		**IL-6**	8902 (7854−10,267)	10,581 (9426−11,230)	0.02
		**TNF-α**	914 (747−1592)	980 (739−1691)	0.70
	**anti-CD3/CD28**	**IFN-γ**	24,241 (11,720−33,673)	8422 (2585−13,867)	0.03
		**IL-2**	362 (195−438)	305 (54−566)	0.60
		**TNF-α**	4405 (2160−7150)	3554 (2290−6226)	0.77
**HS**	**PHA**	**IFN-γ**	18,351 (8902−23,093)	18,568 (9905−25,120)	1.00
		**IL-2**	829 (534−1070)	973 (219−2191)	0.57
		**TNF-α**	3379 (2584−4544)	3147 (1908−5117)	0.51
	**LPS**	**IL-1**	2179 (1663−3234)	2328 (1724−3578)	0.76
		**IL-6**	6107 (5523−6738)	6837 (6186−7136)	0.10
		**TNF-α**	678 (558−851)	836 (634−1313)	0.09

Young (YG) and older (OG) group. Median (Q1–Q3). * Significant differences determined by Wilcoxon rank sum test. FBS, fetal bovine serum; HS, autologous human serum; PHA, phytohemagglutinin; LPS, lipopolysaccharide; IFN-γ: interferon γ; IL-2: interleukin 2; TNF-α: tumor necrosis factor α; IL-1β: interleukin 1β; IL-6: interleukin 6; N, number.

## References

[1] Flegal K.M., Carroll M.D., Ogden C.L., Curtin L.R. (2010). Prevalence and trends in obesity among US adults, 1999–2008. JAMA.

[2] Moller N., O’Brien P., Nair K.S. (1998). Disruption of the relationship between fat content and leptin levels with aging in humans. J. Clin. Endocrinol. Metab..

[3] Villareal D.T., Apovian C.M., Kushner R.F., Klein S. (2005). Obesity in older adults: Technical review and position statement of the American Society for Nutrition and NAASO, The Obesity Society. Am. J. Clin. Nutr..

[4] Douziech N., Seres I., Larbi A., Szikszay E., Roy P.M., Arcand M., Dupuis G., Fulop T. (2002). Modulation of human lymphocyte proliferative response with aging. Exp. Gerontol..

[5] Meydani S.N., Endres S., Woods M.M., Goldin B.R., Soo C., Morrill-Labrode A., Dinarello C.A., Gorbach S.L. (1991). Oral (n-3) fatty acid supplementation suppresses cytokine production and lymphocyte proliferation: Comparison between young and older women. J. Nutr..

[6] Karlsson E.A., Beck M.A. (2010). The burden of obesity on infectious disease. Exp. Biol. Med. (Maywood).

[7] Milner J.J., Beck M.A. (2012). The impact of obesity on the immune response to infection. Proc. Nutr. Soc..

[8] Tanaka S., Isoda F., Ishihara Y., Kimura M., Yamakawa T. (2001). T lymphopaenia in relation to body mass index and TNF-alpha in human obesity: Adequate weight reduction can be corrective. Clin. Endocrinol. (Oxf.).

[9] Nieman D.C., Henson D.A., Nehlsen-Cannarella S.L., Ekkens M., Utter A.C., Butterworth D.E., Fagoaga O.R. (1999). Influence of obesity on immune function. J. Am. Diet. Assoc..

[10] Yang H., Youm Y.H., Vandanmagsar B., Rood J., Kumar K.G., Butler A.A., Dixit V.D. (2009). Obesity accelerates thymic aging. Blood.

[11] Falagas M.E., Kompoti M. (2006). Obesity and infection. Lancet Infect. Dis..

[12] Dorshkind K., Montecino-Rodriguez E., Signer R.A. (2009). The ageing immune system: Is it ever too old to become young again?. Nat. Rev. Immunol..

[13] Gruver A.L., Hudson L.L., Sempowski G.D. (2007). Immunosenescence of ageing. J. Pathol..

[14] Gubbels Bupp M.R. (2015). Sex, the aging immune system, and chronic disease. Cell. Immunol..

[15] Solana R., Tarazona R., Gayoso I., Lesur O., Dupuis G., Fulop T. (2012). Innate immunosenescence: Effect of aging on cells and receptors of the innate immune system in humans. Semin. Immunol..

[16] Nikolich-Zugich J. (2008). Ageing and life-long maintenance of T-cell subsets in the face of latent persistent infections. Nat. Rev. Immunol..

[17] Hunsche C., Hernandez O., De la Fuente M. (2016). Impaired Immune Response in Old Mice Suffering from Obesity and Premature Immunosenescence in Adulthood. J. Gerontol. A Biol. Sci. Med. Sci..

[18] Ahmed T., Das S.K., Golden J.K., Saltzman E., Roberts S.B., Meydani S.N. (2009). Calorie restriction enhances T-cell-mediated immune response in adult overweight men and women. J. Gerontol. A Biol. Sci. Med. Sci..

[19] Hager K., Machein U., Krieger S., Platt D., Seefried G., Bauer J. (1994). Interleukin-6 and selected plasma proteins in healthy persons of different ages. Neurobiol. Aging.

[20] Wei J., Xu H., Davies J.L., Hemmings G.P. (1992). Increase of plasma IL-6 concentration with age in healthy subjects. Life Sci..

[21] Tussing-Humphreys L., Pini M., Ponemone V., Braunschweig C., Fantuzzi G. (2011). Suppressed cytokine production in whole blood cultures may be related to iron status and hepcidin and is partially corrected following weight reduction in morbidly obese pre-menopausal women. Cytokine.

[22] Garcia C., Feve B., Ferre P., Halimi S., Baizri H., Bordier L., Guiu G., Dupuy O., Bauduceau B., Mayaudon H. (2010). Diabetes and inflammation: Fundamental aspects and clinical implications. Diabetes Metab..

[23] Liu C., Feng X., Li Q., Wang Y., Li Q., Hua M. (2016). Adiponectin, TNF-alpha and inflammatory cytokines and risk of type 2 diabetes: A systematic review and meta-analysis. Cytokine.

[24] Gameiro C.M., Romão F., Castelo-Branco C. (2010). Menopause and aging: Changes in the immune system—A review. Maturitas.

[25] Verma S., Hussain M.E. (2017). Obesity and diabetes: An update. Diabetes Metab. Syndr..

